# Healthcare discrimination and factors associated with gender-affirming healthcare avoidance by transgender women and transgender men in Thailand: findings from a cross-sectional online-survey study

**DOI:** 10.1186/s12939-023-01843-4

**Published:** 2023-02-13

**Authors:** Nachale Boonyapisomparn, Natthaporn Manojai, Pimwarat Srikummoon, Walaithip Bunyatisai, Patrinee Traisathit, Nontiya Homkham

**Affiliations:** 1 Astraea Lesbian Foundation for Justice, New York, USA; 2 School of Business and Graduate Studies, Trinity Washington University, Washington, DC USA; 3 The Foundation of Transgender Alliance for Human Rights, Bangkok, Thailand; 4 Mplus Foundation, Chiang Mai, Thailand; 5 Department of Statistics, Faculty of Science, Chiang Mai University, Chiang Mai, Thailand; 6 Data Science Research Center, Department of Statistics, Faculty of Science, Chiang Mai University, Chiang Mai, Thailand; 7 Research Center in Bioresources for Agriculture, Industry and Medicine, Department of Statistics, Faculty of Science, Chiang Mai University, Chiang Mai, Thailand; 8 Faculty of Public Health, Thammasat University, Pathumthani, Thailand

**Keywords:** LGBT, Transgender women, Transgender men, Discrimination, Healthcare service

## Abstract

**Background:**

Although discriminatory experiences of transgender people seeking healthcare services have been well-documented in several studies, differentiating those experiences based on gender identity/expression and related factors has been limited. The aim of this study was to compare the characteristics, experiences, attitude, and expectation toward accessing healthcare service and healthcare providers of transgender women and transgender men in Thailand.

**Methods:**

A cross-sectional study was conducted from October 2017 to March 2018. The data were collected from transgender women and transgender men aged ≥ 18 years old who lived in Thailand using online platform via different websites and Facebook pages of local transgender group. Binary logistic regression was used to identify the factors related to the study outcomes.

**Results:**

Of 186 transgender people who responded to the questionnaire and were eligible for the study, 73.7% (95% confidence interval [CI] = 66.7–79.8) were transgender women and 26.3% (95% CI = 20.2–33.3) were transgender men. Transgender women were more likely to seek general healthcare from non-traditional healthcare services (crude odds ratio [cOR] = 4.28; 95% CI = 1.55–11.81; *P* = 0.005), buy hormone treatment from non-traditional healthcare services (cOR = 3.89; 95% CI = 1.18–12.83; *P* = 0.026), and receive healthcare counseling from non-traditional healthcare providers (cOR = 5.16; 95% CI = 1.42–18.75; *P* = 0.013) than transgender men. According to the results of applying a multivariable model, transgender respondents who did not know that gender-affirming healthcare services existed in Thailand were more unwilling to receive counseling from gender-affirming healthcare providers than those who did (adjusted odds ratio = 3.70; 95% CI = 1.11–12.36; *P* = 0.033).

**Conclusions:**

The findings from this cross-sectional study indicate that transgender women are more likely than transgender men to receive general healthcare and hormone treatment from non-traditional healthcare services and buy hormone treatment without a physician’s supervision. We also found approximately 15% of transgender individuals who did not receive gender-affirming counseling services. Continuing to improve access to care for the transgender community, increasing public relations channels may encourage transgender people to access more healthcare services.

**Supplementary Information:**

The online version contains supplementary material available at 10.1186/s12939-023-01843-4.

## Background

Stigma- and discrimination-driven inequities suffered by transgender people (e.g., insufficient or nonexistent rights and protection, inadequate healthcare services, poor educational and professional opportunities, victimization, rejection, and isolation) [1–5] can lead to mental problems, negative health outcomes, and negative behavioral consequences (e.g., depression, psychological distress, suicidal ideation, poor self-worth, substance use, and taking sexual risks) [6–13]. It has previously been reported that transgender individuals also experience health inequality in the treatment of HIV, mental health problems, and drug and alcohol use compared to cisgender individuals [8, 14–18]. Several researchers have also reported that transgender individuals experience a higher prevalence of discrimination compared to gay, lesbian, and bisexual individuals, which adversely affects their health and well-being throughout their lives [19–21]. Hughto et al. [6] clearly explained how social stigmatization (the process of labeling, stereotyping, and marginalizing as a form of social control) can impact transgender people's well-being. It can occur at the structural, interpersonal, and individual levels and is a fundamental cause of adverse health outcomes among the transgender population.

The number of transgender individuals seems to have been underestimated due to social barriers against recognizing gender identities outside of the usual gender binary model (a person can only be a biological male identifying as a man or a biological female identifying as a woman) and the reluctance of transgender people to disclose their gender identity [22, 23]. Thus, the amount of healthcare data available on the transgender population is limited as only gender binarism has historically been applied (with a few exceptions) for data collection in population-based studies. Emerging findings on transgender people's differential healthcare experiences have revealed important disparities among gender identity/expression subgroups. Transgender men were twice as likely as transgender women to postpone needed healthcare due to anticipated discrimination [24, 25]. Although non-binary individuals are less likely to delay receiving healthcare than transgender women [25], they are also significantly less likely to report being treated with respect by healthcare providers after disclosure of their transgender identity compared to cisgender individuals [26]. To the best of our knowledge, no studies have been conducted on the healthcare experiences of transgender people in Thailand.

It has been reported that poor healthcare and/or discriminatory treatment of lesbian, gay, bisexual, transgender, and others (LGBT +) individuals in the healthcare system are primary associated factors of mental health issues [27]. Both direct and indirect discrimination by healthcare providers toward LGBT + individuals were reported in a previous study in Thailand including (1) inappropriate disclosure of private information, (2) a lack of awareness and competence when addressing LGBT + health issues, (3) applying unequal standards of care to LGBT + versus cisgender individuals, (4) characterizing LGBT + status as a mental illness, and (5) outright refusing to treat LGBT + people [27].

The Tangerine Community Health Center in Bangkok, which opened in November 2015, is the first clinic in Thailand to provide trans-specific healthcare and counseling [28]. Currently, more gender-affirming healthcare services (i.e., access to counseling, hormone treatment, gender-affirming surgery, and other transgender-related surgical procedures) are being established in both public and private hospitals, as well as transgender-exclusive non-profit organizations [29]. Moreover, the Thai Ministry of Public Health has reported on transgender people’s access to HIV and sexually transmitted infections (STIs) services and found that around 15% of healthcare providers had negative attitudes toward transgender women who sought HIV-related services. These negative experiences were circulated and communicated within the community, and consequentially, many transgender people delay or avoid seeking healthcare services [30].

We hypothesized that experience and stigma issues are associated with the willingness to seek and the expectation concerning healthcare services among the transgender population in this study. Previously, the findings from studies in several countries (i.e., Argentina, Australia, Jamaica, the US, and Thailand) have revealed that transgender individuals who experience stigma and discrimination when accessing healthcare services avoid/delay accessing general healthcare or seek non-traditional healthcare services [27, 31–37]. Gender identity is an interesting variable. Researchers in the United States found that the transgender men were 1.29 times more likely than transgender women to attend a transgender-specific care provider [33]. Hormone usage and the pathway to access it are also interesting. Previously, researchers in the US and Thailand reported that transgender women were less likely than transgender men to attend a hormone management program and transgender-specific care [33, 37, 38]. In addition, in previous studies in the US and Canada, the authors also found that the avoidance of accessing healthcare services by transgender individuals resulted from the lack of healthcare providers who understand transgender concerns [26, 39]. Although the discriminatory experiences of transgender people seeking healthcare services have been well-documented in several studies, differentiating them based on gender identity/expression and related factors has been limited. The experience, attitude, and expectation toward accessing healthcare services and healthcare providers might be different between transgender women and transgender men, and to the best of our knowledge, no study has been conducted to examine the relationship between discrimination experiences and the attitude and expectation toward accessing healthcare service in this context in Thailand. The aim of this study was to compare the characteristics, experiences, attitude, and expectation of transgender women and transgender men in Thailand toward accessing healthcare services and healthcare providers. We also investigated the effects of characteristics, gender identification, and discrimination experiences on gender-affirming healthcare avoidance and the expectation that healthcare workers/staff can fulfill transgender healthcare needs.

## Methods

### Study context

Some of the questions in the National Transgender Discrimination Survey in the USA conducted by the National Center for Transgender Equality and the National Gay Lesbian Task Force in 2011 were adopted and then translated into the Thai context for the online questionnaire [40]. The questionnaire consisted of 5 main sections, including (1) demographic characteristics, (2) the attitudes of transgender individuals toward healthcare, (3) experience with accessing traditional and non-traditional healthcare services, (4) experience with gender-affirming healthcare services in Thailand, and (5) the expectation of transgender individuals when accessing healthcare services.

### Participants and setting

A cross-sectional observational study was conducted from October 2017 to March 2018. The data were collected from transgender women and transgender men aged ≥ 18 years old who lived in Thailand via an online questionnaire.

### Data collection and measurements

The online questionnaire used in this study was developed according to the CHERRIES reporting guidelines [41]. An information sheet comprising a description of the survey design (i.e., the title of the research, a short background piece about the study, the target population, and the aims of the study) and a yes/no question asking for consent to participate (“Would you like to participate in this study?”) was provided in the first page of the questionnaire. All of the participants gave their consent after having been informed about the aim of the study as well as their right to refuse to participate.

The information sheet, informed consent, and study protocol were reviewed and approved by the Bachelor of General Studies (BGS) Institutional Review Board at Trinity Washington University. The participants who completed the online self-reporting questionnaire were anonymized before inclusion in the analyses. To reach the two transgender populations in Thailand, the online questionnaire was distributed through the websites and Facebook pages of local transgender and lesbian, gay, bisexual, and transgender (LGBT) organizations such as the Foundation of Transgender Alliance for Human Rights, the Sisters Foundation, as well as transgender men groups. The study information was revalidated before being compiled and used for statistical analysis. The revalidation procedures included (1) checking the response rate and unique site visitors (monitored by matching data using the Stata program), (2) deleting multiple entries from the same individual using the Stata program, and (3) preparing the dataset for statistical analysis using the Stata program.

### Study variables

The demographic characteristics included transgender identity, age, monthly income, nationality, religion, region of residence, highest educational achievement, employment status, relationship status, and sex work experience in the previous year.

The attitudes of transgender individuals toward healthcare were assessed by using five scale-rated items: (1) transgender health concerns are different from cisgender ones, (2) seeking healthcare providers who understand transgender concerns is easy, (3) there is a high chance of experiencing discrimination in the healthcare setting, (4) avoiding needed medical attention/care and treatment, and (5) buying hormones or other medicines without a doctor’s supervision. The answer for each item was ranked using a three-point Likert scale (“disagree”, “neutral”, or “agree”).

The experiences of transgender individuals in relation to five areas of healthcare services, including general healthcare services, hormone treatment, gender-affirming surgery, other transgender-related surgical procedures, healthcare counseling, and HIV testing from traditional or non-traditional healthcare services were also assessed. Traditional healthcare services were defined as public hospitals, private hospitals, and healthcare clinics whereas non-traditional healthcare services included pharmacies, Thai traditional medicine clinics, non-profit organizations, transgender-exclusive non-profit organizations, the Internet, and online social networks.

The next part of the questionnaire comprised two questions asking about gender-affirming healthcare services: (1) the perception of whether gender-affirming healthcare services for transgender individuals exist in Thailand and (2) unwillingness to receive healthcare counseling from gender-affirming healthcare providers. Gender-affirming healthcare services are specifically for helping transgender individuals physically transition (i.e., counseling, hormone treatment, gender-affirming surgery, and other transgender-related surgical procedures).

In addition, the expectation of transgender individuals when accessing healthcare services and healthcare workers/staff concerning their transgender healthcare needs was assessed via three questions: (1) access to healthcare staff/workers who are sensitive to transgender health needs and concerns, (2) access to doctors and healthcare professionals who are knowledgeable about transgender health needs and concerns, and (3) providing transgender-specific healthcare services. The answers were ranked from “1 = very low”, “2 = low”, “3 = neutral”, “4 = high”, and “5 = very high”. High and low levels of expectation concerning whether healthcare workers/staff can fulfill transgender healthcare needs were defined as > 12 and ≤ 12 points, respectively. Furthermore, experience of unfair gender discrimination was defined as when doctors or healthcare providers did not respect their gender identity, used the wrong pronoun, or refused to administer healthcare services. All of the study variables are shown in Supplementary Fig. 1.

### Sample size calculation

The sample size was calculated based on the following formula [42]:

$$n=\frac{{Z}_{\alpha /2}^{2}\bullet P(1-P)}{{e}^{2}},$$

where n is the required sample size. ${Z}_{\alpha /2}$ is set as 1.96 for $\alpha$ = 0.05; and $P$ is the prevalence of transgender men and transgender women who have received hormones from non-traditional healthcare services set as 94% according to that used in a previous study about cross-sex hormone use and general health and well-being among transgender people in Thailand [43]. The accepted error in this study was set as e = 0.035 and the confidence interval (CI) was set as 95%. Thus, at least 177 participants were required for the study.

### Statistical analysis

All data analyses were performed using Stata version 15 [44]. In the bivariate analyses, Chi-squared tests and Fisher’s exact tests were used to compare the demographic characteristics, attitudes toward healthcare services, receiving healthcare services from traditional and non-traditional healthcare services, and having and never having experienced unfair gender discrimination between transgender men and transgender women.

Binary logistic regression was used to identify the factors related to the study outcomes, including accessing healthcare services at non-traditional health services, unwillingness to receive counseling via gender-affirming healthcare services, and high expectation that healthcare workers/staff understand transgender healthcare needs. The potential risk factors related to the outcomes in the univariable analysis with *P*-value < 0.25 or clinically relevant variables were included in the multivariable analysis with backward elimination. Kachen et.al [33] suggested that compared to transgender men, transgender women were more likely to be unwilling to receive counseling from gender-affirming healthcare providers and had a higher level of expectation that healthcare workers/staff can fulfill transgender healthcare needs. Therefore, transgender identity (transgender men or transgender women) was also retained as an adjusted factor in the multivariable models. Finally, the model fitness was checked by using the Hosmer–Lemeshow goodness-of-fit test. *P*-value < 0.05 was considered statistically significant.

## Results

### Comparison of the demographic characteristics of transgender men and transgender women

Of 186 transgender people who responded to the questionnaire and were eligible for the analysis, 49 (26.3%; 95% confidence interval [CI] = 20.2–33.3) were transgender men and 137 (73.7%; 95% CI = 66.7–79.8) were transgender women. Their socio-demographic characteristics are reported in Table 1. More than 70% were over 25 years old and most of them were Thai. 49.0% (95% CI = 34.4–63.7) of transgender men and 53.3% (95% CI = 44.6–61.9) of transgender women were employed full-time. The monthly income of most of the transgender women was below 15,001 baht while half of the transgender men had a monthly salary of 15,001–30,000 baht (*P* = 0.026). Moreover, more of the transgender men lived with partners than transgender women (67.3% (95% CI = 52.5–80.1) versus 27.0% (95% CI = 19.8–35.3); *P* < 0.001), and 17.5% (95% CI = 11.6–24.9) of transgender women had been sex workers whereas none of transgender men had (Table 1).

**Table 1:** Characteristics of the transgender men and transgender women participants (n = 186)

Characteristics	Transgender Men		Transgender Women		*P*
	**(n = 49)**		**(n = 137)**		
	**n (%)**	**95% CI**	**n (%)**	**95% CI**	
Age					0.650^a^
< 25 years	12 (25.0)	13.6–39.6	29 (21.8)	15.1–29.8	
≥ 25 years	36 (75.0)	60.4–86.4	104 (78.2)	70.2–84.9	
N/A	1	-	4	-	
Nationality					0.057^b^
Thai	46 (93.9)	83.1–98.7	136 (99.3)	96.0–100.0	
Others	3 (6.1)	1.3–16.9	1 (0.7)	0.0–4.0	
Religion					0.228^b^
Buddhist	43 (87.8)	75.2–95.4	128 (93.4)	87.9–97.0	
Others	6 (12.2)	4.6–24.8	9 (6.6)	3.0–12.1	
Region of residence					0.299^a^
Bangkok	19 (38.8)	25.2–53.8	42 (30.7)	23.1–39.1	
Others	30 (61.2)	46.2–74.8	95 (69.3)	60.9–76.9	
Highest educational level					0.629^a^
< A bachelor's degree	33 (67.3)	52.5–80.1	87 (63.5)	54.9–71.6	
≥ A bachelor's degree	16 (32.7)	19.9–47.5	50 (36.5)	28.4–45.1	
Employment status					0.707^a^
Full-time	24 (49.0)	34.4–63.7	73 (53.3)	44.6–61.9	
Self-employed/business owner	10 (20.4)	10.2–34.3	21 (15.3)	9.7–22.5	
Part-time/student/unemployed	15 (30.6)	18.3–45.4	43 (31.4)	23.7–39.9	
Monthly income (baht)					0.026^a^
< 15,001	17 (34.7)	21.7–49.6	57 (42.9)	34.3–51.7	
15,001–30,000	26 (53.1)	38.3–67.5	43 (32.3)	24.5–41.0	
> 30,000	6 (12.2)	4.6–24.8	33 (24.8)	17.7–33.0	
N/A	-		4		
Relationship status					< 0.001^a^
Single	16 (32.7)	19.9–47.5	100 (73.0)	64.7–80.2	
Partner	33 (67.3)	52.5–80.1	37 (27.0)	19.8–35.3	
Sex work experience					0.002^a^
No	49 (100.0)	-	113 (82.5)	75.1–88.4	-
Yes	0 (0.0)	-	24 (17.5)	11.6–24.9	-

N/A Not applicable, 95% CI 95% confidence interval

^a^*P*-value derived by using a Chi-squared test

^b^*P*-value derived by using Fisher’s exact test

### Attitudes of transgender men and transgender women toward healthcare

**Fig. 1:**
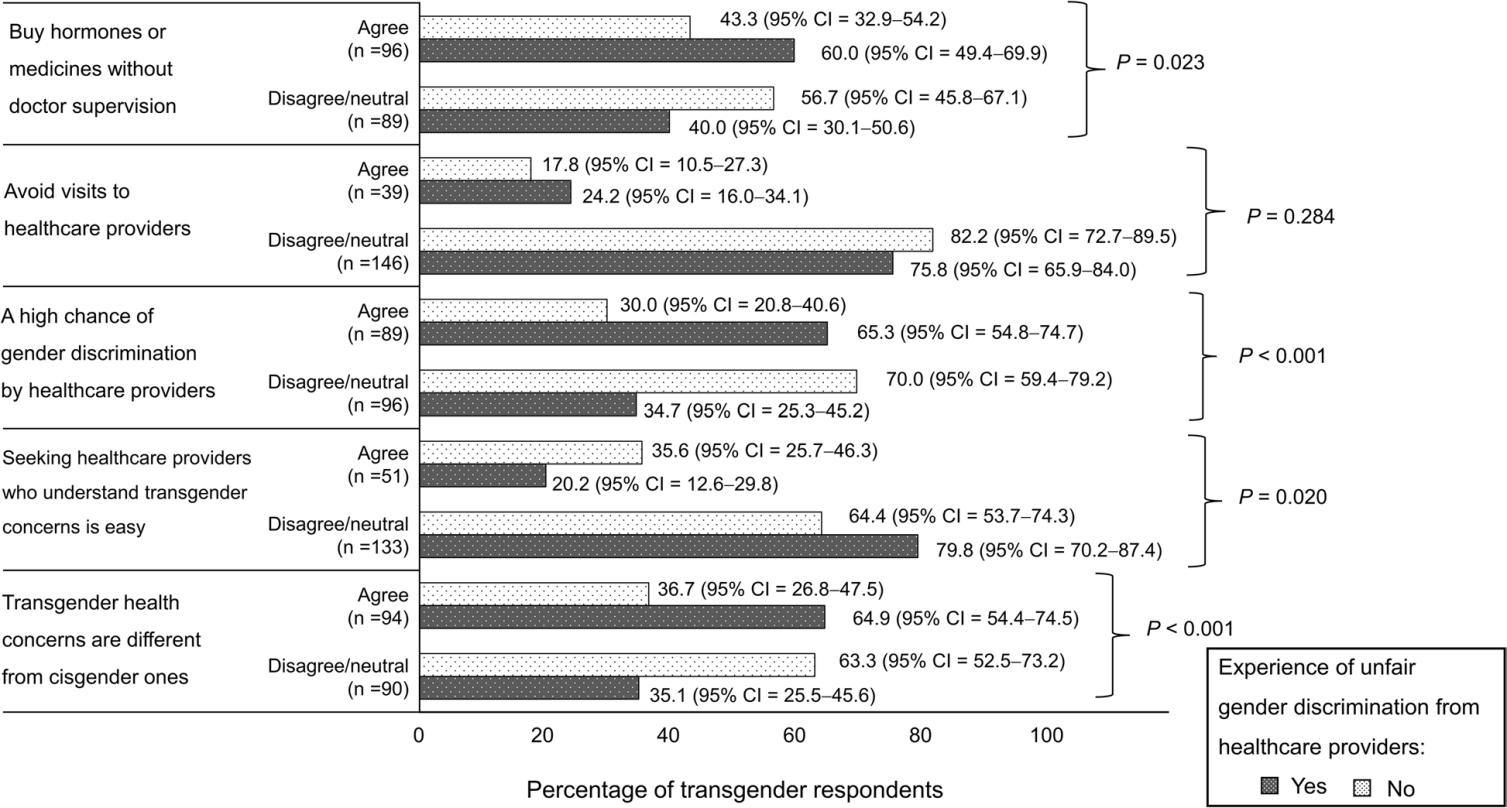
Attitudes toward healthcare among transgender individuals who have or have never experienced discrimination from healthcare providers. 95% CI, 95% confidence interval; *P*, *P*-value derived by using a Chi-squared test

The majority of the transgender individuals (51.4%; 95% CI = 63.4–88.2) had experienced unfair gender discrimination from healthcare providers and were significantly more likely to agree that transgender health concerns are different from those of cisgender individuals than those who had never experienced discrimination (64.9% (95% CI = 54.4–74.5) versus 36.7% (95% CI = 26.8–47.5); *P* < 0.001). This was also the case for seeking healthcare providers who understand transgender health concerns is easy (79.8% (95% CI = 70.2–87.4) versus 64.4% (95% CI = 53.7–74.3); *P* = 0.020). In addition, transgender respondents who had experienced discrimination from healthcare providers were more likely to agree that transgender people have a higher chance of experiencing unfair gender discrimination from healthcare providers than cisgender individuals (65.3% (95% CI = 54.8–74.7) versus 30.0% (95% CI = 20.8–40.6); *P* < 0.001). In terms of attitude toward buying hormones and medicine, transgender respondents who had experienced discrimination from healthcare providers were more likely to buy hormones and medicines without a doctor’s supervision than transgender respondents who had never experienced discrimination (60.0% (95% CI = 49.4–69.9) versus 43.3% (95% CI = 32.9–54.2); *P* = 0.023) (Fig. 1).

Table 2 summarizes the attitudes of the 49 transgender men and 137 transgender women toward healthcare. Half of the transgender women agreed that transgender health concerns are different from cisgender ones, while half of the transgender men either disagreed with or had no opinion about this issue. Most of the respondents in both groups disagreed that seeking healthcare providers who understand transgender health issues is easy (77.6% (95% CI = 63.4–88.2) and 69.9% (95% CI = 60.9–76.9), respectively). Most of the transgender women agreed that transgender people had a higher chance of experiencing unfair gender discrimination from healthcare providers than cisgender people (51.8%; 95% CI = 43.1–60.4), while most of the transgender men disagreed (61.2%; 95% CI = 46.2–74.8). Meanwhile, 16.3% (95% CI = 7.3–29.7) of transgender men and 22.6% (95% CI = 15.9–30.6) of transgender women avoided visiting healthcare providers when feeling unwell because of their transgender identity. In addition, most of the transgender women (66.4%; 95% CI = 57.9–74.3) and a few of the transgender men (12.2%; 95% CI = 4.6–24.8) bought hormones or medicines without a doctor’s supervision (*P* < 0.001).

**Table 2:** Attitudes toward healthcare (n = 186)

Attitude	Total		Transgender Men		Transgender Women		*P*
	**n (%)**	**95% CI**	**n (%)**	**95% CI**	**n (%)**	**95% CI**	
Transgender health concerns are different from cisgender ones							0.085
Disagree/neutral	90 (48.7)	41.2–56.1	29 (59.2)	44.2–73.0	61 (44.8)	36.0–53.3	
Agree	95 (51.3)	43.9–58.8	20 (40.8)	27.0–55.8	75 (55.2)	46.0–63.3	
Seeking healthcare providers who understand transgender concerns is easy							0.304
Disagree/neutral	133 (71.9)	64.8–78.2	38 (77.6)	63.4–88.2	95 (69.9)	60.9–76.9	
Agree	52 (28.1)	21.8–35.2	11 (22.4)	11.8–36.6	41 (30.1)	22.4–38.3	
A high chance of gender discrimination by healthcare providers							0.117
Disagree/neutral	91 (51.6)	44.4–59.3	30 (61.2)	46.2–74.8	66 (48.2)	39.6–56.9	
Agree	90 (48.4)	41.2–56.1	19 (38.8)	25.2–53.8	71 (51.8)	43.1–60.4	
Avoid visits to healthcare providers							0.352
Disagree/neutral	147 (79.0)	72.9–85.0	41 (83.7)	70.3–92.7	106 (77.4)	69.4–84.1	
Agree	39 (21.0)	15.4–27.7	8 (16.3)	7.3–29.7	31 (22.6)	15.9–30.6	
Buy hormones or medicine without doctor supervision							< 0.001
Disagree/neutral	89 (47.8)	40.7–55.6	43 (87.8)	75.2–95.4	46 (33.6)	25.7–42.1	
Agree	97 (52.2)	45.0–59.8	6 (12.2)	4.6–24.8	91 (66.4)	57.9–74.3	

*P P*-value derived by using a Chi-squared test, 95% CI 95% confidence interval

### Healthcare received from traditional and non-traditional healthcare providers by transgender men and transgender women

**Fig. 2:**
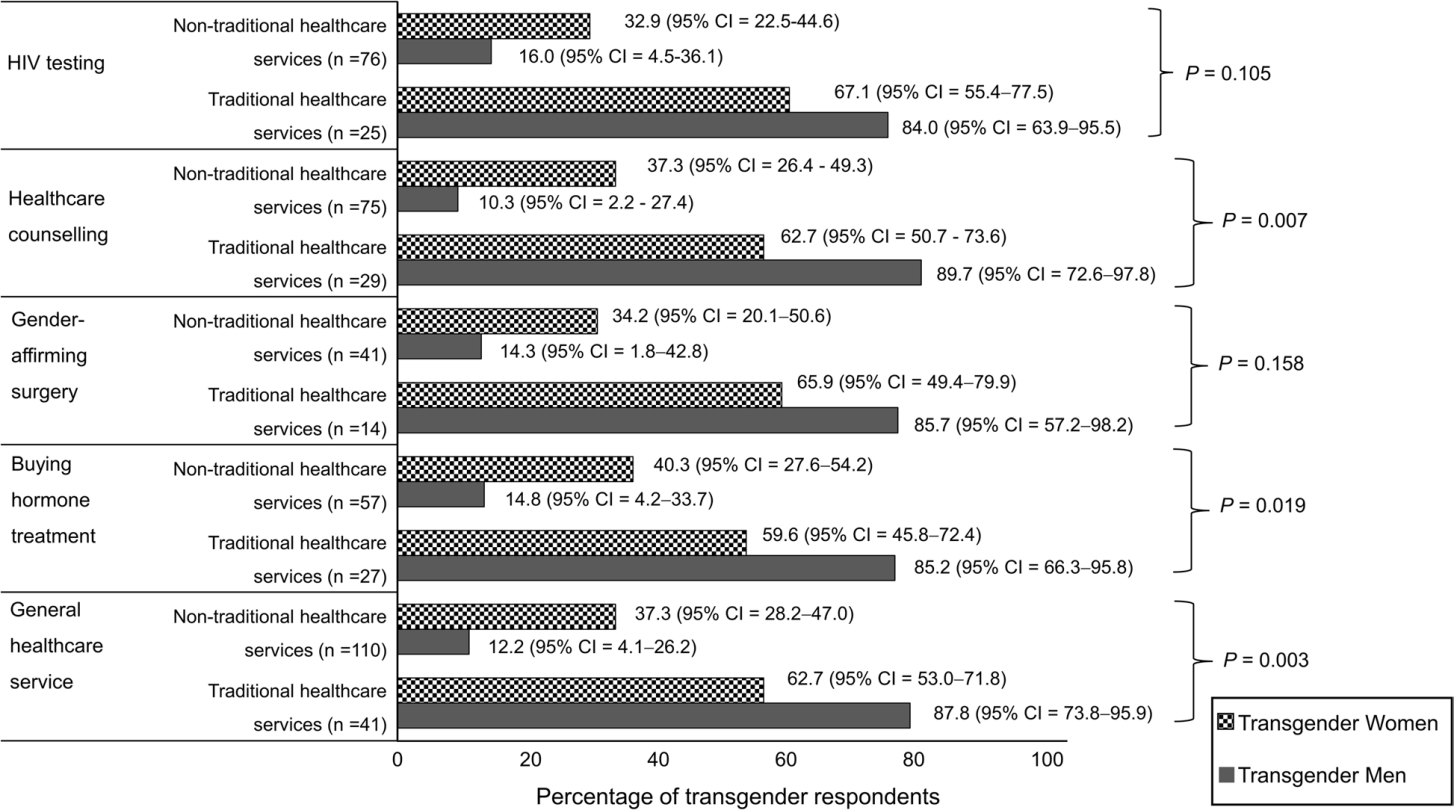
Healthcare services received from traditional and non-traditional health providers. 95% CI, 95% confidence interval;* P*, *P-*value derived by using a Chi-squared test

Figure 2 shows a comparison of transgender men and transgender women who had received healthcare from traditional and non-traditional healthcare services in the previous 12 months. Of the 151 transgender respondents who had received general healthcare from non-traditional healthcare services (i.e., pharmacies, Thai traditional medicine clinics, non-profit organizations, transgender exclusive non-profit organizations, the Internet, and online social media), more transgender women had than transgender men (37.3% (95% CI = 28.2–47.0) versus 12.2% (95% CI = 4.1–26.2); *P* = 0.003). Of the 84 transgender respondents who had received hormone treatment in the past 12 months, significantly fewer transgender men had from non-traditional healthcare services than transgender women (14.8% (95% CI = 4.2–33.7) versus 40.3% (95% CI = 27.6–54.2); *P* = 0.019). Meanwhile, 14.3% (95% CI = 1.8–42.8) of transgender men and 34.2% (95% CI = 20.1–50.6) of transgender women had received gender-affirming surgery and other transgender-related surgical procedures. In addition, significantly more transgender women had received healthcare consultation from non-traditional healthcare services than transgender men (37.3% (95% CI = 26.4–49.3) versus 10.3% (95% CI = 2.2–27.4); *P* = 0.007), while 16% (95% CI = 4.5–36.1) of transgender men and 32.9% (95% CI = 22.5–44.6) of transgender women had undergone HIV testing via non-traditional healthcare services.

### Association between transgender identity and accessing non-traditional healthcare services

The univariable analyses for the association between a transgender individual’s characteristics and accessing healthcare at non-traditional healthcare services (i.e., general healthcare service, buying hormone treatment, gender-affirming surgery, other transgender-related surgical procedures, healthcare counseling, and HIV testing) in the previous year show that transgender identity was only associated with some of them (Supplementary Tables S1, S2, S3, S4 and S5). Table 3 reports a comparison between the transgender identity groups and accessing healthcare services at non-traditional healthcare services in the previous year. Compared to transgender men, transgender women were more likely to receive general healthcare from non-traditional healthcare services (crude odds ratio [cOR] = 4.28; 95% CI = 1.55–11.81; *P* = 0.005), while buying hormone treatment from non-traditional healthcare services was statistically significant (cOR = 3.89; 95% CI = 1.18–12.83; *P* = 0.026), as was receiving healthcare counseling from non-traditional healthcare services (cOR = 5.16; 95% CI = 1.42–18.75; *P* = 0.013) (Table 3).

**Table 3:** Association between transgender identity and healthcare services accessing at non-traditional healthcare services

Healthcare Services	Accessing Non-Traditional Healthcare Services		
	**n/N (%)**	**cOR (95% CI)**	***P***
General healthcare services (n = 151)			0.005
Transgender men (ref.)	5/41 (12.2)	1	
Transgender women	41/110 (37.3)	4.28 (1.55 – 11.81)	
Buying hormone treatment (n = 84)			0.026
Transgender men (ref.)	4/27 (14.8)	1	
Transgender women	23/57 (40.4)	3.89 (1.18 – 12.83)	
Gender-reassignment surgery and other transgender-related surgical procedures (n = 55)			0.176
Transgender men (ref.)	2/14 (14.3)	1	
Transgender women	14/41 (34.2)	3.11 (0.60 – 16.12)	
Healthcare counseling (n = 104)			0.013
Transgender men (ref.)	3/29 (10.3)	1	
Transgender women	28/75 (37.3)	5.16 (1.42 – 18.75)	
HIV testing (n = 101)			0.116
Transgender men (ref.)	4/25 (16.0)	1	
Transgender women	25/76 (32.9)	2.57 (0.79 – 8.35)	

cOR Crude odds ratio, 95% CI 95% confidence interval, ref. Reference group, *P P*-value derived by using a binary logistic regression (Wald test)

### Factors associated with the unwillingness to receive counseling from a gender-affirming healthcare service

In terms of gender-affirming healthcare services, most of the transgender respondents knew that gender-affirming healthcare services exist (80.1%; 95% CI = 73.6–85.6). The results of the multivariable logistic regression analysis to ascribe factors associated with the unwillingness to receive counseling from gender-affirming healthcare providers are provided in Table 4. According to the results, transgender respondents who disagreed with buying hormones or medicines without doctor supervision (adjusted odds ratio [aOR] = 5.53; 95% CI = 1.50–20.42; *P* = 0.010) and transgender respondents who did not know that gender-affirming healthcare services exist in Thailand (aOR = 3.70; 95% CI = 1.11–12.36; *P* = 0.033) were more likely to be unwilling to receive counseling from gender-affirming healthcare providers compared to their respective comparison group after adjusting for gender identity (Table 4). However, the unwillingness to receive counseling from gender-affirming healthcare providers was not significantly different between transgender women transgender and men. Moreover, the model was a good fit according to using the Hosmer–Lemeshow goodness-of-fit test (*P* = 0.557).

**Table 4:** Factors associated with the unwillingness to receive counseling from gender-affirming healthcare providers

Variables	Unwillingness	Univariable analysis		Multivariable analysis	
**Characteristics**	**n (%)**	**cOR (95% CI)**	***P***	**aOR (95% CI)**	***P***
Age			0.703		
< 25 years (ref.)	4/24 (16.7)	1			
≥ 25 years	10/74 (13.5)	0.78 (0.22 – 2.78)			
Region of residence			0.854		
Bangkok	4/31 (12.9)	0.89 (0.25 – 3.11)			
Others (ref.)	10/70 (14.3)	1			
Religion			0.864		
Buddhist (ref.)	14/95 (14.7)	1			
Others	1/8 (12.5)	0.83 (0.09 – 7.32)			
Highest education level			0.529		
< A bachelor's degree	10/61 (16.4)	1.45 (0.46 – 4.63)			
≥ A bachelor's degree (ref.)	5/42 (11.9)	1			
Employment status			0.552		
Self-employed/business owner (ref.)	1/16 (6.3)	1			
Full-time	9/51 (17.6)	3.21 (0.37 – 27.84)			
Part-time/student/unemployed	5/36 (13.9)	2.42 (0.26 – 22.83)			
Relationship status			0.618		
Partner (ref.)	7/42 (16.7)	1			
Single	8/61 (13.1)	0.75 (0.25 – 2.28)			
Experience of unfair gender discrimination from healthcare providers			0.005		
Yes (ref.)	2/54 (3.7)	1			
No	13/49 (26.5)	9.39 (1.98 – 44.49)			
Monthly income (baht)			0.859		
< 15,001	7/47 (14.9)	1.58 (0.29 – 8.41)			
15,001 – 30,000	5/34 (14.7)	1.55 (0.27 – 8.94)			
> 30,000 (ref.)	2/20 (10.0)	1			
Sex work experience			0.593		
No (ref.)	14/92 (15.2)	1			
Yes	1/11 (9.1)	0.56 (0.07 – 4.75)			
Gender identity			0.277		0.862
Transgender men (ref.)	5/23 (21.7)	1		1	
Transgender women	10/80 (12.5)	0.51 (0.16 – 1.70)		0.90 (0.26—3.09)	
**Gender-affirming healthcare service**					
Perception of whether gender-affirming healthcare services for transgender individuals exist in Thailand			0.096		0.033
Known (ref.)	7/68 (10.3)	1		1	
Unknown	8/35 (22.9)	2.58 (0.85 – 7.88)		3.70 (1.11—12.36)	
**Attitudes toward healthcare**					
Buy hormones or medicine without doctor supervision			0.019		0.010
Agree (ref.)	4/58 (6.9)	1		1	
Disagree/neutral	11/45 (24.4)	4.37 (1.28 – 14.92)		5.53 (1.50—20.42)	
Transgender health concerns are different from cisgender ones			0.055		
Agree (ref.)	5/58 (8.6)	1			
Disagree/neutral	10/44 (22.7)	3.12 (0.98 – 9.97)			
Seeking healthcare providers who understand transgender concerns is easy			0.455		
Agree (ref.)	5/26 (19.2)	1.57 (0.48 – 5.15)			
Disagree/neutral	10/76 (13.2)	1			
A high chance of gender discrimination by healthcare providers			0.056		
Agree (ref.)	4/52 (7.7)	1			
Disagree/neutral	11/51 (21.6)	3.30 (0.97 – 11.23)			
Avoid visits to healthcare providers			0.679		
Agree (ref.)	3/25 (12.0)	1			
Disagree/neutral	12/78 (15.4)	1.33 (0.34 – 5.20)			

cOR Crude odds ratio, aOR Adjusted odds ratio, 95% CI 95% confidence interval, ref. Reference group, *P P*-value derived by using a binary logistic regression (Wald test)

### Factors associated with a high level of expectation that healthcare workers/staff can fulfill transgender healthcare needs

Table 5 reports the univariable logistic regression analysis concerning factors associated with a high level of expectation that healthcare workers/staff can fulfill transgender healthcare needs. Transgender individuals who have never been sex workers remained significantly more likely to have a high level of expectation of this compared to those who are or have been sex workers (aOR = 4.60; 95% CI = 1.23–17.19; *P* = 0.023) after adjusting for gender identity and monthly income (Table 5). However, a high level of expectation that healthcare workers/staff can fulfill transgender healthcare needs was not significantly associated with either of these factors. The model was a good fit, as confirmed by using the Hosmer–Lemeshow goodness-of-fit test (*P* = 0.756).

**Table 5:** Factors associated with a high level of expectation that healthcare worker/staff can fulfill transgender healthcare needs

Variables	High expectation	Univariable analysis		Multivariable analysis	
**Characteristics**	**n/N (%)**	**cOR (95% CI)**	***P***	**aOR (95% CI)**	***P***
Age			0.216		
< 25 years	39/40 (97.5)	3.71 (0.47 – 29.65)			
≥ 25 years (ref.)	126/138 (91.3)	1			
Region of residence			0.135		
Bangkok	58/60 (96.7)	3.22 (0.69 – 14.95)			
Others (ref.)	108/120 (90.0)	1			
Religion			0.768		
Buddhist (ref.)	153/168 (91.1)	1			
Others	14/15 (93.3)	1.37 (0.17 – 11.24)			
Highest educational level			0.145		
< A bachelor's degree (ref.)	104/117 (88.9)	1			
≥ A bachelor's degree	63/66 (95.5)	2.63 (0.72 – 9.61)			
Employment status			0.529		
Self-employed/business owner	28/30 (93.3)	1.96 (0.38 – 10.13)			
Full-time	89/96 (92.7)	1.78 (0.59 – 5.38)			
Part-time/student/unemployed (ref.)	50/57 (87.7)	1			
Relationship status			0.949		
Single (ref.)	103/113 (91.2)	1			
Partner	64/70 (91.4)	1.04 (0.36 – 3.00)			
Experience of unfair gender discrimination from healthcare providers			0.261		
Yes (ref.)	87/93 (93.6)	1			
No	79/89 (88.8)	0.54 (0.19 – 1.57)			
Monthly income (baht)			0.119		0.139
< 15,001 (ref.)	63/73 (86.3)	1		1	
15,001 – 30,000	63/67 (94.0)	2.50 (0.74 – 8.42)		2.33 (0.72 – 7.49)	
> 30,000	38/39 (97.4)	6.03 (0.74 – 49.28)		5.29 (0.61 – 46.17)	
Sex work experience			0.033		0.023
Yes (ref.)	19/24 (79.2)	1		1	
No	148/159 (93.1)	3.54 (1.11 – 11.33)		4.60 (1.23 – 11.19)	
Gender identity			0.674		0.222
Transgender men (ref.)	44/49 (89.8)	1		1	
Transgender women	123/134 (91.8)	1.27 (0.42 – 3.87)		2.30 (0.61 – 8.71)	
**Gender-affirming healthcare service**					
Perception of whether gender-affirming healthcare services for transgender individuals exist in Thailand			0.535		
Unknown (ref.)	31/35 (88.6)	1			
Known	136/148 (91.9)	1.46 (0.44 – 4.86)			
**Attitudes toward healthcare**					
Buy hormones or medicine without doctor supervision			0.497		
Disagree/neutral (ref.)	79/88 (89.8)	1			
Agree	88/95 (92.6)	1.43 (0.51 – 4.04)			
Transgender health concerns are different from cisgender ones			0.148		
Disagree/neutral (ref.)	78/88 (88.6)	1			
Agree	89/94 (94.7)	2.28 (0.75 – 6.99)			
Seeking healthcare providers who understand transgender concerns is easy			0.503		
Disagree/neutral (ref.)	120/132 (90.9)	1			
Agree	47/50 (94.0)	1.57 (0.42 – 5.82)			
A high chance of gender discrimination by healthcare providers			0.155		
Disagree/neutral (ref.)	83/94 (88.3)	1			
Agree	84/89 (94.4)	2.23 (0.74 – 6.71)			
Avoid visits to healthcare providers			0.664		
Disagree/neutral (ref.)	133/145 (91.7)	1			
Agree	34/38 (89.5)	0.77 (0.23 – 2.54)			

cOR Crude odds ratio, aOR Adjusted odds ratio, 95% CI 95% confidence interval, ref. Reference group, *P P*-value derived by using a binary logistic regression (Wald test)

## Discussion

This cross-sectional study was conducted on transgender women and transgender men in Thailand to compare their characteristics, as well as experiences, attitude, and expectation toward accessing healthcare services, and investigate their effects on the gender-affirming healthcare avoidance and expectation that healthcare workers/staff can fulfill transgender healthcare needs. Our findings show that experience of unfair gender discrimination from healthcare providers toward transgender individuals was relatively higher (51.4%; 95% CI = 63.4–88.2) than that reported in previous studies in the US and Australia (approximately 10–41%) [31–35]. The discrimination experience when accessing healthcare services was also higher compared to the general population in Thailand (5%) [45]. Our findings also show an association between experience of discrimination from healthcare providers and buying hormones or medicines without a physician’s supervision. This is consistent with findings from previous studies suggesting that transgender individuals avoided or delayed seeking medical care due to their concern about discrimination from the healthcare providers [36, 46]. The results of previous studies also suggest that the experience of stigma and discrimination from accessing healthcare services might affect them to avoid/delay accessing general healthcare or to switch using healthcare services from non-traditional healthcare services [31–37].

Our results show that transgender women were fourfold more likely to buy hormone treatment from non-traditional healthcare services (cOR = 3.89; 95% CI = 1.18–12.83) than transgender men. This is consistent with the outcomes of previous studies in Thailand and other countries inferring that transgender women were less likely to attend a hormone management program and transgender-specific care than transgender men [33, 37, 38]. A previous survey in Australia showed that 31% of transgender individuals thought that accessing hormones was too difficult, 17% thought that the cost of a doctor’s appointment was too expensive and 16% were unable to find a doctor to prescribe hormones [31]. Meanwhile, most feminizing hormones can be purchased over the counter without a physician’s prescription in Thailand [47], which might have influenced the decision of transgender individuals to obtain hormone treatment without the physician’s prescription.

Although the transgender men had a significantly higher monthly income than the transgender women in our study, income was not associated with the decision to access healthcare among the transgender individuals. The cost of accessing the healthcare service might be one of the factors related to avoidance or delays in assessing general healthcare. The findings from a previous study in the US infer that transgender men are more likely to avoid accessing healthcare services than transgender women due to the cost (34.8% versus 25.0%). In addition, there is also a barrier when transgender individuals try and attain health insurance. The outcomes of previous studies suggest an association between avoiding accessing healthcare services and health insurance issues among transgender populations [36, 48]. In Thailand, gender-affirming surgery and other related healthcare services are non-reimbursable in both the public and private health insurance systems, so transgender individuals will have to pay for it themselves [49]. However, the costs and access to health insurance for transgender individuals were not included in this study. Further study to explore the influences of these factors on the decision to access traditional healthcare services would be interesting.

Other than the barriers due to the cost of healthcare services and health insurance, other barriers might influence the decision to access healthcare from traditional healthcare providers, such as affordability, service accessibility, the healthcare provider, and the waiting period [50]. The outcomes from a previous study in the US indicate that transgender men are 1.29 times more likely than transgender women to visit a transgender-specific care provider [33]. This is consistent with our study, in which transgender women were significantly more likely to receive general healthcare service from non-traditional healthcare providers (cOR = 4.28; 95% CI = 1.55–11.81) and received healthcare counseling from non-traditional healthcare providers (cOR = 5.16; 95% CI = 1.42–18.75) than transgender men in the previous year. A lack of specific knowledge of transgender health, inclusive policies, standards of trans-competent healthcare, and specific healthcare services available for transgender people might produce barriers to accessing traditional healthcare services. We found that more than 70% of transgender individuals agreed that seeking a healthcare provider who understands transgender concerns is not easy (71.9%; 95% CI = 64.8–78.2). Similar to the results of a previous study in the US and Canada, a major barrier to accessing healthcare services among transgender individuals is due to healthcare providers not understanding transgender healthcare issues [26, 39].

Although there are several services in the healthcare setting for transgender people (i.e., access to counseling, hormone treatment, gender-affirming surgery, and other transgender-related surgical procedures), approximately 15% of transgender individuals who participated in our study did not know that gender-affirming healthcare services were available in Thailand. Moreover, those who did not know about the availability of gender-affirming healthcare services were fourfold more likely to avoid counseling (aOR = 3.70; 95% CI = 1.11–12.36). According to the Thai handbook of transgender healthcare services in 2021, the key recommendations for public health policies were to improve access to healthcare for transgender people by including a transgender healthcare service package in the national health coverage, to treat transgender clients with respect and dignity, to provide access to transgender healthcare services and information for everyone across the country, to educate and train healthcare providers of all levels on transgender-competent care and sensitivity, and to conduct more research on and include transgender people’s health needs [51].

Although transgender women were more likely to buy hormone treatment and receive healthcare counseling from non-traditional healthcare services than transgender men, gender identity was not associated with the willingness to receive counseling from a gender-affirming healthcare service. However, the transgender respondents who disagreed with or were neutral about buying hormones or medicines without a doctor’s supervision were sixfold more unwilling to receive counseling from a gender-affirming healthcare provider than those who agreed (aOR = 5.53; 95% CI = 1.50–20.42). Although the specific reasons for their unwillingness were not considered in the present study, it would be beneficial to investigate them in the future. In addition, since we adjusted the model to account for gender identity, there might be other confounding factors to be investigated in further study.

There are some limitations of this study. First, the data collection using online platforms such as websites and Facebook pages of local transgender and LGBT organizations and groups might have contributed to selection bias. A transgender individual without access to the Internet would not have been able to fill in this online survey questionnaire. In addition, the non-probability sampling methods and self-reporting used in the study limit the generalizability of the results. We used a cross-sectional study design and thus, the recognition of causality cannot be inferred. Second, the relatively small sample size in our study might not have truly covered the demographics, diversity, and variations in the transgender community, such as gender, age, nationality, religion, and city of residence. Finally, we did not include some variables that might have contributed to or have been consequences of healthcare avoidance among the transgender populations, such as the cost, right to treatment, reasons for avoidance, physical and mental health issues, the side effects of long-term hormones usage, and the difference between receiving standard and non-standard healthcare. Therefore, a well-design future study (e.g., a longitudinal study) with a larger sample size and incorporating other factors should be conducted to determine the factors influencing healthcare access and the consequences of accessing healthcare among the transgender population.

## Conclusion

The findings from this cross-sectional study indicate that transgender women are more likely than transgender men to receive general healthcare and hormone treatment from non-traditional healthcare services and buy hormone treatment without a physician’s supervision. Although gender-affirming healthcare services exist in Thailand and are established in public and private hospitals, and transgender-exclusive non-profit organizations, we still found that approximately 15% of transgender individuals did not know about gender-affirming counseling services and were less likely to seek counseling via gender-affirming healthcare services. In addition, transgender women were significantly more likely than transgender men to receive hormone treatment and gender general healthcare services from non-traditional healthcare services. Continuing to improve access to healthcare for the transgender community, increasing public relations channels about how to access and locate gender-affirming healthcare services may encourage transgender people, especially transgender women, to access them more often.

## Supplementary Information

**Additional file 1.**

**Additional file 2: Supplementary Table S1.** Factors associated with receiving general healthcare services from non-traditional healthcare services.

**Additional file 3: Supplementary Table S2.** Factors associated with buying hormone treatment from non-traditional healthcare services.

**Additional file 4: Supplementary Table S3.** Factors associated with received sex reassignment surgery and other transgender-related surgery from non-traditional healthcare services.

**Additional file 5: Supplementary Table S4.** Factors associated with received healthcare counseling from non-traditional healthcare services.

**Additional file 6: Supplementary Table S5.** Factors associated with HIV test from non-traditional healthcare services.

## Data Availability

The datasets used and/or analyzed during the current study are not publicly available due to lack of previous approval to share data publicly. The datasets used and/or analyzed during the current study can be made available through a data-sharing agreement with the corresponding author on reasonable request.

## Abbreviations

aOR: Adjusted odds ratio
BGS: Bachelor of General Studies
cOR: Crude odds ratio
LGBT: Lesbian, gay, bisexual and transgender
